# Ultrasound-activated miR-195-5p/shikonin nanobubbles remodel immunosuppressive microenvironment *via* immunogenic cell death to potentiate PD-1/PD-L1 blockade in hepatocellular carcinoma

**DOI:** 10.1016/j.mtbio.2025.102216

**Published:** 2025-08-18

**Authors:** Yandi Tan, Qiao Xu, Yezi Chen, Yun Liu, Chaoqi Liu, Xinwu Cui, Yun Zhao

**Affiliations:** a Department of Medical Ultrasound, Tongji Hospital, Tongji Medical College and State Key Laboratory for Diagnosis and Treatment of Severe Zoonotic lnfectious Diseases, Huazhong University of Science and Technology, Wuhan, 430030, China; b Hubei Key Laboratory of Tumor Microenvironment and Immunotherapy, China Three Gorges University, Yichang, China; c Department of Ultrasound Imaging, The First College of Clinical Medical Science, China Three Gorges University, Yichang, 443008, China

**Keywords:** Shikonin, miR-195-5p, Lipid nanobubbles, Ultrasound, Tumour

## Abstract

Despite the transformative clinical impact of programmed cell death ligand 1/programmed death 1 (PD-1/PD-L1) blockade in hepatocellular carcinoma (HCC), therapeutic efficacy remains limited by the tumor immunosuppressive microenvironment (TIME), with objective response rates persistently below 20 %. To address this critical clinical challenge, we engineered ultrasound (US)-responsive lipid nanobubbles (NBs) co-encapsulating microRNA (miR)-195-5p and shikonin (SK) (designated miR-195-5p/SK-NBs), a dual-functional platform designed to synergize PD-L1 suppression with immunogenic cell death (ICD). The NBs exhibited tumor-selective accumulation through passive and active targeting mechanisms while maintaining biosafety. US-triggered microbubble destruction enabled localized miR-195-5p delivery, achieving PD-L1 downregulation and functionally recapitulating PD-1/PD-L1 blockade. Concurrently, SK—a naphthoquinone compound repurposed as a potent ICD inducer—overcame the systemic toxicity and solubility limitations through NBs-mediated delivery, triggering robust release of damage-associated molecular patterns, including adenosine triphosphate, surface-exposed calreticulin, and secreted high-mobility group box 1 protein. In subcutaneous HCC models, miR-195-5p/SK-NBs reprogrammed the TIME by enhancing splenic lymphocyte proliferation, increasing cytotoxic CD8^+^ T cell infiltration and IFN-γ production, and increasing proportion and cytotoxic activity of cytotoxic T lymphocyte. The reversed TIME ultimately improved the antitumor efficacy of anti–PD-1 antibody immunotherapy. The therapeutic superiority stems from coordination between PD-1/PD-L1 blockade and ICD-mediated immune activation, presenting a promising strategy for hepatocellular carcinoma treatment.

## Graphical abstract

**Figure:**
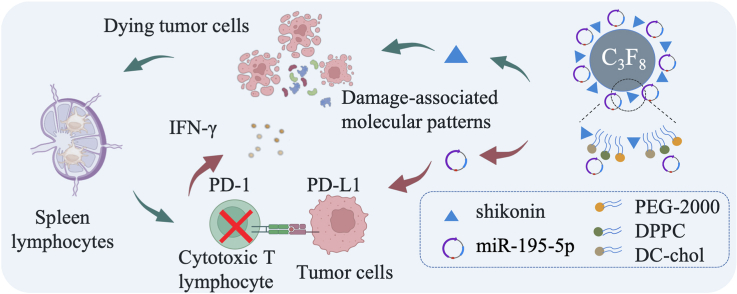

## 1 Introduction

Liver cancer, primarily hepatocellular carcinoma (HCC), represents one of the most formidable therapeutic challenges in contemporary oncology, ranking sixth globally in incidence and third in cancer-related deaths [1,2]. Programmed death 1/programmed cell death ligand 1 (PD-1/PD-L1) blockade has revolutionized the clinical management of HCC [3]. However, clinical data indicate that the objective response rates of representative agents remain suboptimal, with pembrolizumab (18.3 %), nivolumab (15.0 %), and camrelizumab (14.7 %) all demonstrating response rates below 20 % [4]. Enhancing the therapeutic efficacy of immunotherapies persists as a pivotal clinical challenge in this field. The tumor immunosuppressive microenvironment (TIME) facilitates immune evasion through multifaceted mechanisms, rendering TIME remodeling a pivotal scientific issue [5,6] (see Table 1).

**Table 1:** Mass ratio of ingredients.

	DPPC	DSPE-PEG2000	DC-Chol	SK
NBs	5	2	0.5	0
miR-195-5p-NBs	5	2	2	0
SK-NBs	5	2	0.5	0.25
miR-195-5p/SK-NBs	5	2	2	0.25

Immunogenic cell death (ICD) not only induces direct tumor cell cytotoxicity, but also activates the immune system by enhancing antigen production, recognition, procession, and immune cells activation, ultimately leading to tumor elimination [7,8]. During ICD, damage-associated molecular patterns (DAMPs) are released, which include the surface exposure of calreticulin (CRT), the excretion of high-mobility group box 1 protein (HMGB1), and the extracellular release of adenosine triphosphate (ATP). These signals promote the maturation of dendritic cells, which subsequently initiate immune response by presenting antigens to cytotoxic T lymphocytes, thereby targeting and eliminating both primary and metastatic tumor cells [9,10]. Shikonin (SK), a well-studied naphthoquinone compound, is a key bioactive component derived from Lithospermum erythrorhizon. This plant, traditionally referred to as "zicao" in Chinese medicine, has been utilized for over two millennia for its therapeutic properties [11]. SK exhibits multiple properties of an ideal ICD inducer [12]: it efficiently triggers programmed cell death, resists drug-efflux mechanisms, downregulates cancer-associated pro-inflammatory transcription factors, and directly targets metastasized cells. Additionally, it shows negligible suppression of anti-tumorigenic immune cells. While doxorubicin is a well-established inducer of ICD, emerging evidence demonstrates that SK exhibits superior potency in triggering ICD and augmenting the antitumor efficacy of dendritic cell-based cancer vaccines compared to doxorubicin [13]. However, the severe systemic toxicity and poor solubility of SK limit its clinical application [14].

Currently, a growing number of nanoplatforms have been explored for drug loading and delivery to reduce premature clearance and off-target effects of therapeutic agents [15,16]. Nevertheless, significant scientific challenges remain unresolved, including insufficient tumor penetration and cellular uptake, as well as inadequate spatiotemporal control over payload release [17]. To address these limitations, we have used an ultrasound (US)-responsive nanoliposomal nanobubbles (NBs) platform. This system leverages US targeted microbubble destruction (UTMD) to achieve non-invasive, site-specific, and temporally controlled release of therapeutic agents at the tumor site, significantly reducing systemic exposure compared to conventional carriers. In addition, UTMD-mediated cavitation dynamics disrupts both vascular and tumor cell membranes, thereby facilitating enhanced tissue penetration and cellular uptake [18]. Lipid NBs demonstrate high loading capacity for hydrophobic SK, primarily through encapsulation within their phospholipid bilayer and hydrophobic core, leveraging the compound's lipophilic characteristics [19].

Simultaneously, microRNA (miR)-195-5p was surface-conjugated on NBs *via* streptavidin-biotin linkage. miR-195-5p is a tumor-suppressive non-coding RNAs that targets multiple oncogenes, implicating in migration, invasion, proliferation, and chemoresistance [20]. Studies have demonstrated that tumor tissues exhibit decreased expression of miR-195 alongside elevated PD-L1 levels [21,22]. Mechanistically, miR-195 specifically downregulates PD-L1 expression on tumor cell surfaces, thereby inhibiting the PD-1/PD-L1 signaling axis. This blockade promotes T-cell activation, enhances secretion of interferon (IFN)-γ and tumor necrosis factor (TNF)-α, and ultimately augments anti-tumor immunity [23,24]. Despite their therapeutic potential, miRNAs confront critical challenges, including poor stability in systemic circulation, insufficient targeting specificity, and suboptimal therapeutic efficacy [25]. The NB platform efficiently protected nucleic acids from nuclease degradation. In addition, UTMD-mediated transient sonoporation markedly increasing gene transfection efficiency [26,27].

**Fig. 1:**
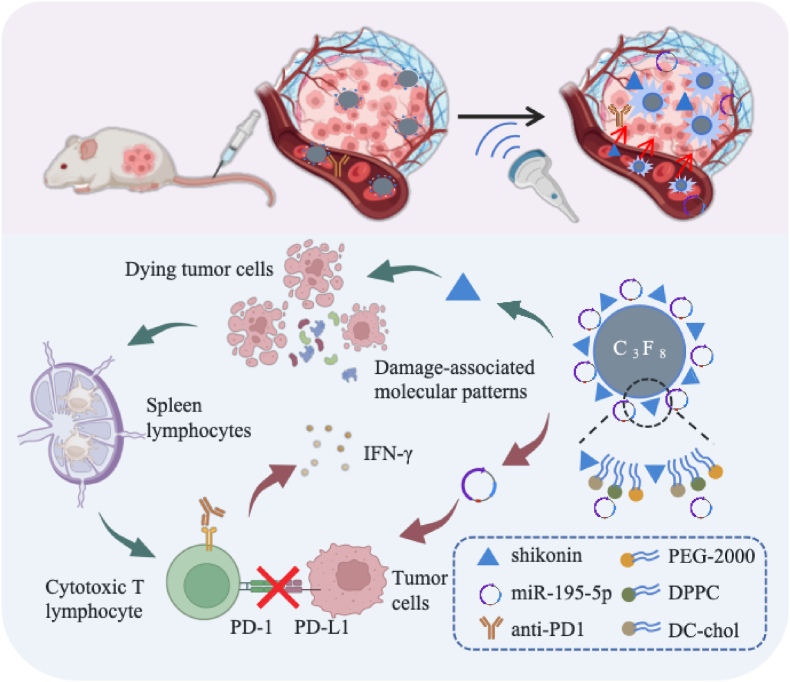
US-responsive lipid NBs co-delivering miR-195-5p and shikonin (miR-195-5p/SK-NBs) were engineered to synergize PD-L1 suppression and ICD for enhanced antitumor immunity. The NBs achieved tumor-targeted accumulation *via* passive/active mechanisms, ensuring biosafety. US-triggered microbubble destruction enabled localized miR-195-5p release, downregulating PD-L1 expression, functionally mimicking PD-1/PD-L1 blockade. Concurrently, NB-mediated SK delivery overcame systemic toxicity, inducing robust ICD with the secretion of CRT, HMGB1, and ATP. In HCC models, miR-195-5p/SK-NBs reprogrammed the TIME, amplifying splenic lymphocyte proliferation, cytotoxic CD8^+^ T cell infiltration, IFN-γ production, proportion and cytotoxic activity of CTL activity, improving the tumor suppression of anti–PD-1 antibody. Created with BioRender.com.

In this study, we engineered lipid NBs co-loaded with miR-195-5p and SK (denoted miR-195-5p/SK-NBs), designed to deliver these therapeutic agents to tumor sites *via* both active and passive targeting mechanisms. Under UTMD, miR-195-5p is efficiently transfected into tumor cells, leading to the downregulation of PD-L1 expression on the tumor cell surface. This downregulation blocks the PD-1/PD-L1 signaling pathway, thereby exerting the therapeutic effects of immune checkpoint inhibitors. Concurrently, SK, an ideal inducer of ICD, penetrates tumor cells more effectively under US irradiation and promotes antigen production and DAMPs release, stimulating immune responses. The miR-195-5p/SK-NBs represent a novel therapeutic platform capable of integrating immune checkpoint inhibition and induction of ICD, thereby providing a promising strategy to remodel the TIME and enhance the efficacy of anti–PD-1 antibody (αPD-1) immunotherapy in HCC (Fig. 1).

## 2 Materials and methods

### 2.1 Cell culture and animals

The cell lines H22, Hepa1-6, and HepG2 were obtained from the China Center for Type Culture Collection (CCTCC, Wuhan, China) and stored at the Tumor Microenvironment and Immune Therapy Laboratory of Three Gorges University. H22 cells were cultured in RPMI-1640 medium (Thermo Fisher Scientific, Waltham, MA, USA), while Hepa1-6 and HepG2 cells were maintained in DMEM (Thermo Fisher Scientific, Waltham, MA, USA). Both media were supplemented with 10 % fetal bovine serum (FBS, Gibco, Australia) and 1 % penicillin/streptomycin (Solarbio Life Sciences, Beijing, China), and cells were incubated at 37 °C in a humidified atmosphere containing 5 % CO_2_. Female BALB/c mice (6–8 weeks) were purchased from the Experimental Animal Center of China Three Gorges University (SYXK 2022-0061). All animal procedures were conducted in strict accordance with the National Institutes of Health Guide for the Care and Use of Laboratory Animals and were approved by the Laboratory Animal Management Committee of China Three Gorges University (ethical approval No.: 202205010X). Humane endpoints were implemented, and animals exhibiting severe clinical conditions were euthanized to prevent unnecessary suffering.

### 2.2 Validation of targeting relationship between miR-195-5p and PD-L1

#### 2.2.1 Construction of the miR-195-5p eukaryotic expression vector

The precursor sequence of miR-195-5p was retrieved from the miRBase database for vector construction. Specific primers containing *Eco*R I and Xho I restriction sites were designed using Primer software, with the following sequences: forward primer 5′-GTCGTATCCAGTGCAGGGTCCGAGGTATTCGCACTGGATACGACGCCAAT-3′ and reverse primer 5′-CGTCGCTAGCAGCACAGAAAT-3’. PCR amplification was performed using mouse normal liver tissue genomic DNA as template. The resulting PCR product was purified and analyzed by 2 % agarose gel electrophoresis. Following *Eco*R I and Xho I double digestion, the target fragment was purified using a commercial purification kit. The eukaryotic expression vector pcDNA 3.1(+) was similarly digested and purified. The insert and vector were ligated using T4 DNA ligase at 16 °C overnight. The ligation product was transformed into DH5α competent cells and cultured at 37 °C for 12–16 h. Positive clones were selected for plasmid extraction, and the recombinant plasmid pcDNA 3.1-miR-195-5p was verified through restriction digestion analysis using *Eco*R I, with pcDNA 3.1(+) as a control. The confirmed plasmid was subsequently sequenced by Shanghai Sangon Biotech Co., Ltd.

#### 2.2.2 Luciferase reporter assay

*Firstly, we constructed the Luciferase Reporter Plasmid:* the 3′-UTR sequence of CD274 (PD-L1) was searched using the NCBI website and analyzed for potential miR-195-5p binding sites through sequence alignment. The identified complementary binding site sequences were synthesized by Shanghai Sangon Biotech Co., Ltd. The luciferase reporter gene fragment was generated through annealing and subsequently ligated into the pMIR-Reporter vector, which had been linearized by restriction enzyme digestion. The recombinant plasmid was transformed into DH5α competent cells, followed by colony selection and plasmid extraction. The constructed luciferase reporter plasmid was verified by DNA sequencing to confirm the correct insertion of the target sequence. Then, Hepa1-6 cells were seeded in 24-well plates (2 × 10^5^ cells per well) and allowed to adhere overnight. Cells were co-transfected with the luciferase reporter plasmid and either miR-195-5p mimics, miR mimic NC, miR-195-5p inhibitor, or miR inhibitor NC. After 48 h of incubation, cells were washed with PBS and lysed using cell lysis buffer (200 μL, 10 min). The lysates were centrifuged at 800 rpm for 3 min to remove cellular debris. For luciferase activity measurement, cell lysate (20 μL) was mixed with luciferase assay reagent (100 μL) in a 96-well black plate. Luminescence was immediately measured using a fluorescence plate reader (BD FACSVerse, USA).

#### 2.2.3 Western blotting

Hepa 1–6 cells were incubated in a 6-well plate (8 × 10^5^ cells per well), and were transfected with pcDNA 3.1(+), miR-195-5p, miR mimic NC, miR-195-5p mimic, miR inhibitor NC, and miR-195-5p inhibitor groups. After 48 h, Cells were washed twice with ice-cold PBS and lysed using RIPA buffer (200 μL) on ice (30 min). Cellular debris was removed by centrifugation (12,000 rpm, 15 min, 4 °C). Protein concentration was quantified using a bicinchoninic acid (BCA) assay kit (Boster Biological Technology, Wuhan, China). Protein samples (100 μL) were denatured by mixing with 5 × Laemmli buffer (25 μL) and heating (100 °C, 10 min). Denatured proteins (30 μg per lane) were resolved by 10 % SDS-PAGE and transferred onto polyvinylidene difluoride membranes using a wet transfer system. Membranes were blocked with 5 % non-fat milk in TBST for 1 h at room temperature, followed by overnight incubation at 4 °C with rabbit anti-PD-L1 polyclonal antibody (1:500 dilution, Wanleibio, Shenyang, China). After three TBST washes, membranes were incubated with HRP-conjugated goat anti-rabbit IgG secondary antibody (1:4000 dilution, Jackson ImmunoResearch Inc., West Grove, PA, USA) for 1 h at room temperature. Protein bands were visualized using enhanced chemiluminescence substrate (Bio-Rad Laboratories) and quantified using Image Lab software (Bio-Rad Laboratories) on a ChemiDoc MP Imaging System.

#### 2.2.4 Flow cytometric analysis

Hepa 1–6 cells were treated as described in Section 2.2.3. Following two washes with ice-cold PBS, cells were incubated with primary PD-L1 antibody (Proteintech, Wuhan, China; 1:200 dilution) in 30 μL reaction volume at ambient temperature for 30 min. After three PBS washes, cells were stained with fluorescence-conjugated secondary antibody (30 μL) under light-protected conditions for 30 min. Fluorescence signal acquisition was performed using a BD FACS Verse flow cytometer (BD Biosciences, San Jose, CA, USA), with subsequent data analysis conducted *via* FlowJo software (v10.8.1, Tree Star Inc.).

### 2.3 Synthesis of various NBs

#### 2.3.1 Empty NBs (N-NBs)

Lipid components, including DPPC, DSPE-PEG2000, and DC-Chol at a mass ratio of 5:2:0.5 were precisely weighed and transferred into a glass bottle. The lipid mixture was completely dissolved in chloroform (15 mL). Subsequently, the organic solvent was evaporated using a rotary evaporator (42 °C, 1 h) to form a thin lipid film. The lipid film was then hydrated with a PBS-glycerol solution (9:1, v/v) in a water bath (45 °C, 1 h) to ensure complete membrane dissolution. The resulting lipid solution was aliquoted into sterilized vials (500 μL per vial) and processed using a gas exchange apparatus. Each vial underwent three cycles of vacuum evacuation (30 s) and perfluoropropane gas infusion (30 s). Finally, the vials were subjected to vigorous mechanical agitation using dental amalgamator (90 s) to generate uniform NBs.

#### 2.3.2 SK-loaded NBs (SK-NBs)

The lipid components, including DPPC, DSPE-PEG2000, DC-Chol, and SK, were precisely weighed at a mass ratio of 5:2:0.5:0.25 and transferred into an amber glass bottle to prevent photodegradation. The subsequent preparation procedures followed the established protocol for NBs synthesis.

#### 2.3.3 MiR-195-5p-loaded NBs (miR-195-5p-NBs)

Cationic NBs were used to synthesise *miR-195-5p-NBs and* prepared by modifying the lipid composition, using DPPC, DSPE-PEG2000, and DC-Chol at a mass ratio of 5:2:2, while maintaining the same preparation protocol as for NBs. For miR-195-5p loading, the constructed plasmid was incubated with cationic NBs (plasmid:NBs = 1 μg: 25 μL) at room temperature (30 min).

#### 2.3.4 MiR-195-5p/SK-NBs

The fabrication process was conducted in two sequential steps. Initially, cationic SK-loaded NBs were prepared by precisely weighing the lipid components-DPPC, DSPE-PEG2000, DC-Chol, and SK-at a mass ratio of 5:2:2:0.25. The mixture was transferred into an amber glass bottle to prevent photodegradation. The subsequent fabrication process followed the established protocol for NBs synthesis. For the final miR-195-5p/SK-NBs preparation, the cationic SK-loaded NBs were incubated with the constructed miR-195-5p plasmid (plasmid:NBs = 1 μg:25 μL). The incubation was carried out at room temperature under light-protected conditions (30 min).

### 2.4 Characterization of NBs

The morphology characteristics of miR-195-5p/SK-NBs were analyzed using optical microscope (Nikon Corporation, Tokyo, Japan) and scanning electron microscope (SEM) (JSM-7500F, JEOL, Tokyo, Japan). The average particle diameter and size distribution were measured using a Zetasizer Nano ZS (Malvern Panalytical). The encapsulation efficiency of SK was quantified using a thin film dialysis. Initially, the maximum absorption wavelength and standard calibration curve of SK were established using UV spectrophotometry. To separate free SK from SK-NBs, miR-195-5p/SK-NBs were dialyzed (6 h) using a dialysis membrane with an external solution of ethanol:PBS (1:1 v/v). The dialyzed NBs were then lysed with methanol, and the SK concentration was determined spectrophotometrically. The encapsulation efficiency was calculated using the following equation:

Encapsulationefficiency(%)=(weightofloadedSK÷weightoftotalSK)×100

The plasmid binding capacity of miR-195-5p-NBs was assessed through agarose gel electrophoresis. Various volumes of NBs (0–28 μL) were incubated with miR-195-5p plasmid (1 μg) at room temperature (30 min). The mixtures were then combined with TAE buffer and 6 × loading buffer to achieve a final volume of 30 μL. Subsequently, aliquots (10 μL) were loaded onto a 1 % agarose gel and electrophoresed (110 V, minutes). The gel was visualized using a gel documentation system to evaluate the plasmid binding efficiency based on electrophoretic mobility shifts.

To evaluate the *in vitro* stability of miR-195-5p/SK-NBs, we first assessed their SK release profile. Two batches of miR-195-5p/SK-NBs were placed in dialysis bags and dialyzed against an ethanol:PBS solution (1:1, v/v). Prior to dialysis, one batch was subjected to ultrasonic irradiation (1 MHz, 0.5 W/cm^2^, 50 % duty cycle, 30 s). At 0, 1, 2, 4, 6, 8, 12, and 24 h, the optical density of the dialysate was measured using a full-wavelength microplate reader. The concentration of released SK was quantified based on a standard curve, and the cumulative release profile of SK from SK-NBs was plotted accordingly. In addition, miR-195-5p/SK-NBs were stored at room temperature and collected at 0, 1, 2, 3, and 4 d. Morphological changes were monitored using an optical microscope, while changes in particle size were measured using a dynamic light scattering particle size analyzer.

### 2.5 Cellular uptake of Dil-labeled NBs and Lipo3000

HepG2 cells were seeded into 6-well plates (8 × 10^5^ cells per well) and divided into two groups: Lipo3000-Dil and NBs-Dil. Dil-Labeled Lipo3000 or NBs were added to the wells at various time points at predetermined time points (2, 4, 8, and 12 h) prior to analysis. At 12 h, cellular uptake of Dil-labeled agents was assessed using an inverted fluorescence microscope and quantified by flow cytometry.

### 2.6 Detection of ICD biomarker *in vitro*

Hepa 1–6 cells were incubated in a 6-well plate (8 × 10^5^ cells per well), and divided into 6 experimental groups: control, N-NBs + US, SK, miR-195-5p-NBs + US, SK-NBs + US, and miR-195-5p/SK-NBs + US. Cells were treated with the respective NBs. For US treatment groups, cells were exposed to US irradiation (1 MHz, 0.5 W/cm^2^, 50 % duty cycle, 30 s). After 24 h of treatment, cell culture supernatants were collected, and ATP levels were measured using an ATP Assay Kit (Solarbio, BC0305). The concentration of HMGB1 protein was determined using a mouse ELISA kit (Solarbio, SEKM-0145), following the manufacturer's instructions. Meanwhile, cells were collected and resuspended in primary anti-CRT antibody (30 μL, 1:200 dilution, Proteintech, Wuhan, China) at room temperature for 30 min. Following three washes with PBS, cells were incubated with fluorescence-conjugated secondary antibody (30 μL) in the dark for 30 min. Then, fluorescence intensity was quantified by flow cytometry.

### 2.7 Suppression of PD-L1 expression *in vitro*

Hepa 1–6 and HepG2 cells were incubated in a 6-well plate (8 × 10^5^ cells per well), and divided into 4 groups: Control, N-NBs + US, miR-195-5p-Lipo3000, and miR-195-5p-NBs + US. After 12 h, cells in the N-NBs + US and miR-195-5p-NBs + US groups were incubated with the respective NBs and exposed to US irradiation (1 MHz, 0.5 W/cm^2^, 50 % duty cycle, 30 s). Cells in the miR-195-5p-Lipo3000 group were transfected with the miR-195-5p plasmid using Lipo 3000 Transfection Reagent (TL301, VAzyme) according to the manufacturer's protocol. The expression level of cell surface PD-L1 was subsequently evaluated using Western blotting (as detailed in Section 2.2.3) and flow cytometry (as detailed in Section 2.2.4).

### 2.8 Evaluation of NBs accumulation in tumors and the main organs

To establish the H22 hepatoma tumor model, a suspension containing H22 cells (1 × 10^6^) in phosphate-buffered saline (50 μL) was subcutaneously injected into the right axillary region of each mouse. When the tumor volume was approximately 100 mm^3^, mice were intravenously injected with Free Dil and Dil-miR-195-5p/SK-NBs. Then, the tumors and main organs (heart, liver, spleen, lung, and kidneys) were also harvested at 24h and imaged by a Living Imaging System (Xenogen, USA).

### 2.9 ICD induction *in vivo*

H22 xenograft–bearing mice with tumors of approximately 100 mm^3^ were randomized into 6 treatment groups: Control, N-NBs + US, free SK, miR-195-5p-NBs + US, SK-NBs + US, and miR-195-5p/SK-NBs + US. Each formulation was administered *via* tail-vein injection on days 1, 4, 7, 10, and 13. The control group received saline (200 μL); the Free-SK group received SK solution (200 μL, 5 μg/mL). NBs groups (N-NBs + US, miR-195-5p-NBs + US, SK-NBs + US, and miR-195-5p/SK-NBs + US) were exposed to US irradiation (1 MHz, 1 W/cm^2^, 50 % duty cycle,120 s) immediately after injection of the respective NBs (200 μL). As our previous studies have demonstrated that the majority of NBs reach the tumor and are cleared within 90 s after injection [28]. Thus, we ensured that NBs ruptured at the tumor site and released the drug locally after 120s irradiation. On Day 14, tumors were collected for analysis of ICD markers.

#### 2.9.1 Quantification of HMGB1 secretion *in vivo*

Fresh tumor tissues were homogenized in ice-cold PBS and centrifuged (at 12,000×*g*, 15 min, 4 °C). Supernatants were collected and analyzed for extracellular HMGB1 levels using a commercial ELISA kit (Cloud-Clone Corp., Wuhan, China) following the manufacturer's protocol. Absorbance was measured at 450 nm using a microplate reader (BioTek Synergy H1).

#### 2.9.2 In vivo analysis of CRT and PD-L1 protein expression

Paraformaldehyde-fixed tumor sections (4 μm thickness) were subjected to immunofluorescence staining. Tissue sections were incubated overnight at 4 °C with primary antibodies against CRT (1:150, Mouse monoclonal, Abcam) or PD-L1 (1:200, Rabbit polyclonal, Cell Signaling Technology), followed by species-matched Alexa Fluor®-conjugated secondary antibodies (1:500, Invitrogen). Nuclei were counterstained with DAPI (5 μg/mL, Sigma-Aldrich). Fluorescence signals were captured using a confocal laser scanning microscope (LSM 880, Zeiss) and quantified using ImageJ software.

### 2.10 Immune system activation *in vivo*

Tumor-bearing mice were treated as described in Section 2.9, and spleen tissues were aseptically harvested and mechanically dissociated into single-cell suspensions to evaluate *in vivo* immune activation. Firstly, we analyzed the proliferation of splenic lymphocytes. Spleens were aseptically harvested and mechanically dissociated into single-cell suspensions. Splenocytes were labeled with 5 μM carboxyfluorescein succinimidyl ester (CFSE; Thermo Fisher Scientific) for 30 min at 37 °C, followed by stimulation with tumor antigens (10 μg/mL) for 48 h. Lymphocyte proliferation was quantified *via* flow cytometry. Then, the proportion of cytotoxic T lymphocytes (CTLs) in splenic lymphocytes was analyzed using FCM. Antigen-primed splenocytes were co-cultured with tumor antigens and IL-2 (20 ng/mL) for 24 h. Cells were stained with FITC-conjugated anti-CD3 (1:100, BioLegend) and APC-conjugated anti-CD8α (1:200, BioLegend) antibodies for 30 min at 4 °C. CTL frequency was determined using FCM, with data analyzed using FlowJo v10.8 software. To verify the cytotoxicity of CTL cells, primed splenocytes (effector cells) were co-cultured with H22 target cells at an effector-to-target (E:T) ratio of 50:1 in 96-well plates. Cytolytic activity was measured using a lactate dehydrogenase (LDH) release assay kit (Beyotime Biotechnology), with absorbance recorded at 490 nm. In addition, Paraffin-embedded tumor sections were immunostained with anti-CD8α (1:200, Abcam) and anti-IFN-γ (1:150, Cell Signaling Technology) antibodies, with DAPI counterstaining for nuclear visualization. Fluorescent signals were quantified using ImageJ software.

### 2.11 Synergistic therapeutic efficacy *in vivo*

When the tumor volume reached approximately 100 mm^3^, H22 hepatoma tumor models were randomly divided into 8 groups (n = 6 per group): Control, N-NBs + US, SK, miR-195-5p-NBs + US, SK-NBs + US, miR-195-5p/SK-NBs + US, αPD-1, and miR-195-5p/SK-NBs + αPD-1 + US groups. Treatment regimens were administered *via* tail vein injection on days 1, 4, 7, 10, and 13. The control group received saline (200 μL); the Free-SK group received SK solution (200 μL, 5 μg/mL); NBs groups (N-NBs + US, miR-195-5p-NBs + US, SK-NBs + US, miR-195-5p/SK-NBs + US, and miR-195-5p/SK-NBs + αPD-1 + US groups) were injected with the respective formulations (200 μL); and both the αPD-1 and miR-195-5p/SK-NBs + αPD-1 groups received αPD-1 (25 μg/mouse). For NBs groups, US (1 MHz, 1 W/cm^2^, 50 % duty cycle,120 s) was applied locally to the tumor immediately after NBs injection to facilitate targeted release.

Tumor dimensions were measured every two days using digital calipers, and volumes were calculated using the formula:

$$\text{Volume}=\left(\text{Length}\times {\text{Width}}^{2}\right)/2$$

24 h after the final treatment, mice were euthanized by cervical dislocation following established protocols. Tumor tissues were harvested, weighed, and photographed. Tumor tissues were divided for subsequent analysis: one portion was fixed in 4 % paraformaldehyde for histological examination (H&E and TUNEL staining).

### 2.12 Biosafety assay of miR-195-5p/SK-NBs

To assess the biosafety profile of the NBs formulations, comprehensive histopathological analysis was performed on major organs (heart, liver, spleen, lungs, and kidneys) through H&E staining. Tissue sections were prepared from paraformaldehyde-fixed specimens and examined under light microscopy for pathological alterations. Additionally, systemic toxicity was monitored by recording body weight changes throughout the treatment period using a precision balance.

### 2.13 Statistical analysis

Statistical analysis was conducted with SPSS software 26.0. Quantitative data are presented as the mean ± standard deviation at least three independent experiments. Between-group comparisons were analyzed using Student's t-test or one-way analysis of variance (ANOVA). (∗P < 0.05, ∗∗P < 0.01). GraphPad Prism 10.0 was employed for graphical representation of data.

## 3 Results and discussion

### 3.1 MiR-195-5p-mediated suppression of PD-L1 expression

**Fig. 2:**
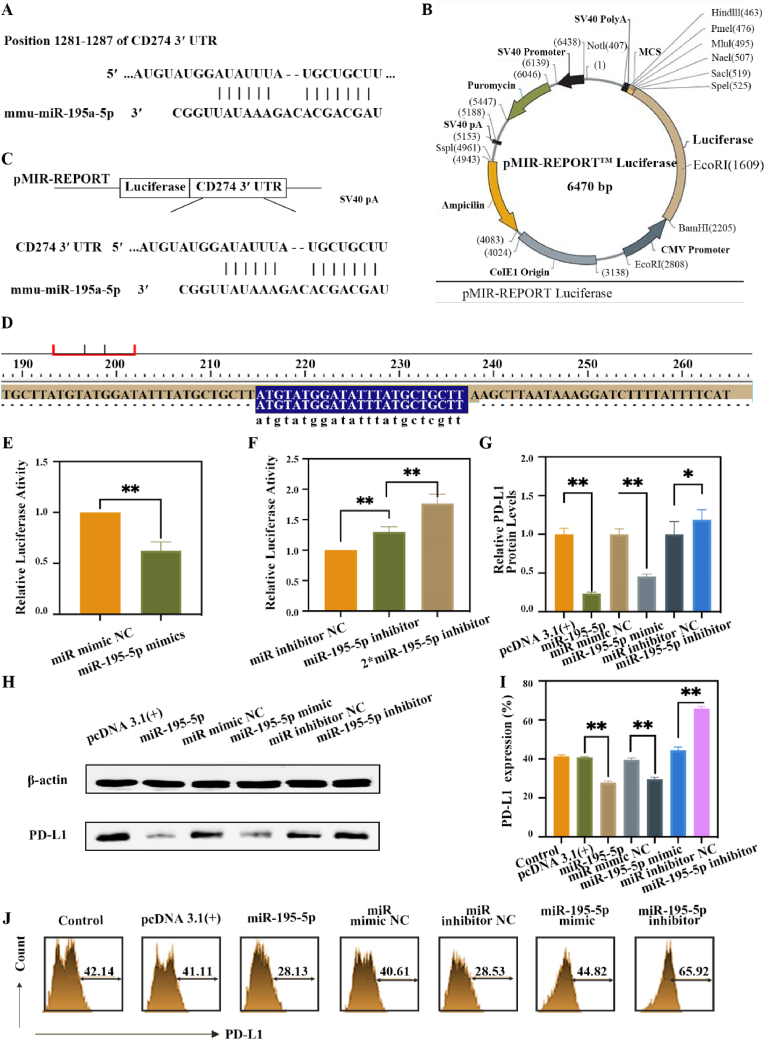
**miR-195-5p-mediated suppression of PD-L1 expression. (**A) Bioinformatic prediction of the miR-195-5p/PD-L1 targeting interaction. (B) Schematic diagram of the pMIR-REPORTER empty vector. (C) Construction strategy of the luciferase reporter plasmid containing the PD-L1 3′-UTR. (D) Sequencing validation of the PD-L1 3′-UTR luciferase reporter plasmid. (E–F) Luciferase reporter assay for miR-195-5p activity in Hepa 1–6 cells, which were co-transfected with miR-195-5p mimics, miR mimic NC, miR-195-5p inhibitor, or miR inhibitor NC for 48 h. Then Hepa 1–6 cells, which were co-transfected with pcDNA 3.1(+), miR-195-5p, miR mimic NC, miR-195-5p mimic, miR inhibitor NC, and miR-195-5p inhibitor for 48h. (G) Quantitative analysis of PD-L1 expression levels across groups by Western blot from panel H. (H) Representative Western blot bands of PD-L1. (I) Statistical analysis of PD-L1 expression from panel J. (J) Flow cytometry plots of PD-L1 expression in Hepa 1–6 cells 48 h post-transfection.

A luciferase reporter plasmid harboring the PD-L1 3′-untranslated region (3′-UTR) was constructed as follows: bioinformatics analysis was used to predict a specific targeting relationship between miR-195-5p and PD-L1, suggesting that miR-195-5p binds to the 3′-UTR of PD-L1to regulate its expression (Fig. 2A). The 3′-UTR sequence of CD274 (PD-L1) was retrieved from the NCBI database, and potential miR-195-5p binding sites were identified through sequence alignment. Using DNAMAN software, we analyzed restriction sites on the pMIR-REPORTER vector and selected Mlu I and *Hin*d III as optimal cleavage sites. A 20–30 bp sequence containing predicted binding sites was selected from the PD-L1 3′-UTR and triplicated to enhance binding affinity, with restriction sites incorporated at both ends. This reporter gene fragment was synthesized by Shanghai Sangon Biotech and subsequently cloned into the pMIR-REPORTER vector using standard molecular cloning techniques (Fig. 2B), generating a luciferase reporter plasmid containing PD-L1 3′-UTR (Fig. 2C). Successful plasmid construction was confirmed by sequencing analysis (Fig. 2D).

To verify whether miR-195-5p can inhibit the expression of PD-L1, the constructed PD-L1 3′-UTR luciferase reporter plasmid was co-transfected into Hepa1-6 cells with miR mimic NC, miR-195-5p mimic, miR inhibitor NC, and miR-195-5p inhibitor, respectively. After 48 h, luciferase activity was measured using a luciferase assay kit. As shown in Fig. 2E and F, compared to the group of miR mimic NC, the luciferase activity of the miR-195-5p mimic group was significantly reduced. Conversely, compared to the group of miR inhibitor NC, the luciferase activity of miR-195-5p inhibitor significantly increased. These results indicate that miR-195-5p specifically targets the 3′-UTR of PD-L1, with the mimic suppressing and the inhibitor enhancing PD-L1 expression. Thus, these findings demonstrate that miR-195-5p specifically targets the 3′-UTR of PD-L1. Our results establish miR-195-5p as a negative regulator of PD-L1 expression through direct 3′-UTR interaction.

To further investigate the regulatory effect of miR-195-5p on PD-L1 expression at the protein level, we performed quantitative analysis using both western blotting and flow cytometry. Hepa1-6 cells were transfected with equal amounts of either pcDNA 3.1(+) (control), miR-195-5p, miR mimic NC, miR-195-5p mimic, miR inhibitor NC, or miR-195-5p inhibitor plasmids. After 48 h of transfection, cells were harvested for protein expression analysis. Western blot analysis (Fig. 2G and H) revealed that transfection with miR-195-5p significantly reduced PD-L1 protein expression compared to the pcDNA 3.1(+) control group (p < 0.01). Consistent with this observation, cells transfected with miR-195-5p mimic exhibited markedly decreased PD-L1 levels relative to the miR mimic NC group (p < 0.01). Conversely, transfection with miR-195-5p inhibitor resulted in a significant upregulation of PD-L1 expression compared to the miR inhibitor NC group (p < 0.05). These findings were further corroborated by flow cytometry analysis of cell surface PD-L1 expression (Fig. 2I and J), which demonstrated a similar pattern of miR-195-5p-mediated PD-L1 regulation. The concordant results from both analytical methods provide compelling evidence that miR-195-5p functions as a negative regulator of PD-L1 expression at the protein level. The observed downregulation of PD-L1 by miR-195-5p and its mimic, coupled with the upregulation by miR-195-5p inhibitor, strongly supports the hypothesis that miR-195-5p directly targets PD-L1 and modulates its expression in HCC cells.

### 3.2 MiR-195-5p/SK-NBs preparation and characterization

**Fig. 3:**
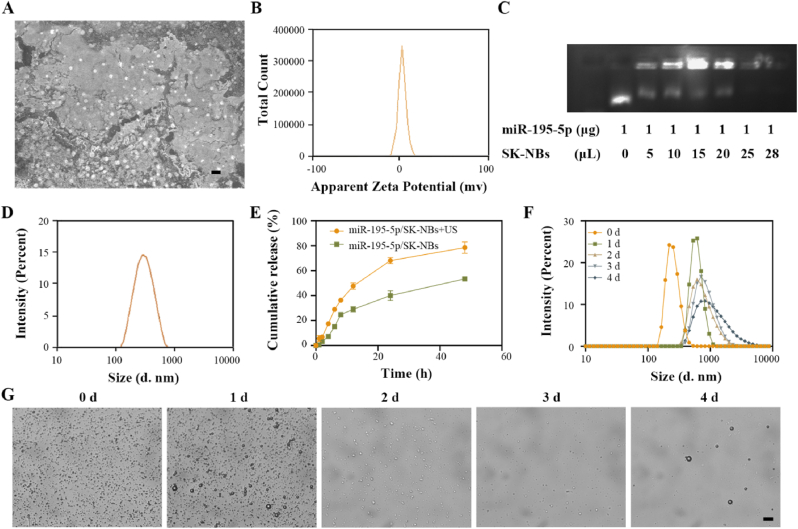
**Characterization of miR-195-5p/SK-NBs.** (A) Morphology of miR-195-5p/SK-NBs analyzed by SEM at 10,000 × magnification. (scale bar = 1 μm) (B) Zeta potential of miR-195-5p/SK-NBs. (C) Agarose gel electrophoresis of plasmid-loaded SK-NBs. (D) Size distribution profile of miR-195-5p/SK-NBs measured by dynamic light scattering. (E) Cumulative SK release from miR-195-5p/SK-NBs over 24 h with or without US treatment. (F) Time-dependent particle changes at 0, 1, 2, 3, and 4 d of miR-195-5p/SK-NBs measured by dynamic light scattering. (G) Representative microscopy images showing morphological changes in miR-195-5p/SK-NBs at 0, 1, 2, 3, and 4 d (scale bar = 10 μm).

SEM (Fig. 3A) revealed that miR-195-5p/SK-NBs exhibited a spherical morphology with uniform size distribution, encapsulated within a lipid shell containing C_3_F_8_ gas. The zeta potential of the NBs was 7.14 ± 1.97 mV (Fig. 3B). Positively charged NBs efficiently loaded negatively charged plasmid DNA; binding‐capacity analysis showed that 25 μL of NBs could effectively complex with 1 μg of plasmid DNA (Fig. 3C). The average particle size was 258.8 ± 3.23 nm with the dispersity index of 0.174, indicating miR-195-5p/SK-NBs had a good dispersity (Fig. 3D). The drug encapsulation efficiency of SK in miR-195-5p/SK-NBs was measured to be 43.29 ± 2.59 %. The simultaneous delivery of hydrophobic SK and hydrophilic miR-195-5p poses formulation challenges due to their divergent physicochemical properties. This was overcome by lipid-phase compartmentalization, which enables co-encapsulation of SK within the phospholipid bilayer through solvent evaporation, while the cationic lipid surface electrostatically binds miR-195-5p. This dual-compartment structure preserves SK bioactivity and protects miRNA from degradation in circulation.

To systematically evaluate the *in vitro* stability of miR-195-5p/SK-NBs, we assessed their SK release kinetics, particle size distribution, and morphological integrity over time. As shown in Fig. 3E, the cumulative release of SK from miR-195-5p/SK-NBs gradually increased over time. In the absence of US, approximately 53.29 ± 1.97 % of SK was released within 24 h. In contrast, SK-NBs exposed to US exhibited a significantly faster release rate, with approximately of SK released within the first 4 h and reaching 78.56 ± 4.50 % by 24 h. These results indicate that US exposure significantly enhances the release efficiency of SK from the NBs. It is suggested that UTMD provides spatiotemporal control to confine payload release to tumor sites, thereby mitigating systemic toxicity and off-target effects. In addition, the particle size (Fig. 3F) and morpohological integrity (Fig. 3G**)** of miR-195-5p/SK-NBs were monitored at 0, 1, 2, 3, and 4 d. Dynamic light scattering analysis revealed a time-dependent increase in the average particle size of miR-195-5p/SK-NBs during storage, indicating gradual physical destabilization, likely due to NBs swelling, fusion, or partical collapse. Consistently, inverted microscopy showed that while freshly prepared miR-195-5p/SK-NBs maintained a uniform spherical morphology with well-defined boundaries, marked deformation and aggregation were observed after 3 and 4 d, suggesting progressive structural degradation. These results demonstrate that SK-NBs possess good short-term stability under physiological conditions. However, structural degradation over time or under US exposure compromises NB integrity, thereby enhancing SK release efficiency.

### 3.3 Cellular uptake of Dil-labeled NBs and Lipo 3000

**Fig. 4:**
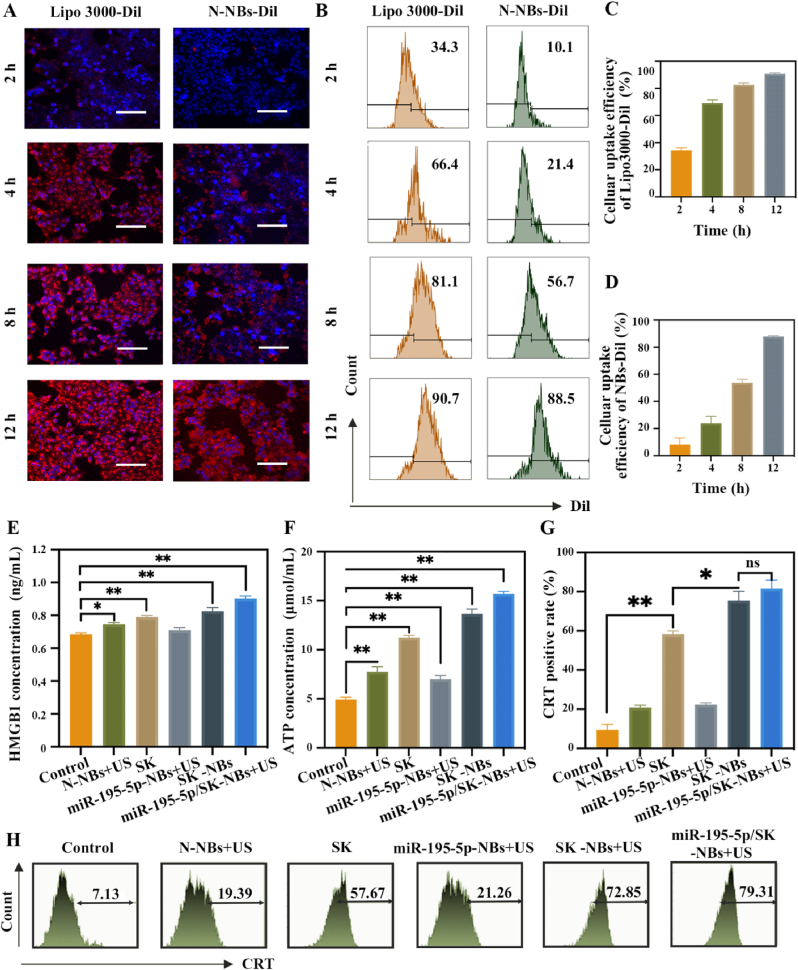
***Invitro* feasibility verification.** (A) Fluorescence microscopy of DiI-labeled Lipo3000 and NBs in HepG2 cells at 0, 4, 8, and 12 h post-incubation (scale bar = 40 μm). (B) Quantification of DiI uptake in HepG2 cells by flow cytometry at 0, 4, 8, and 12 h post-incubation. (C–D) Corresponding flow cytometry results from panel B. Hepa 1–6 were treated with N-NBs + US, SK, miR-195-5p-NBs + US, SK-NBs + US, and miR-195-5p/SK-NBs + US for 24 h. Then, culture supernatants were collected to measure extracellular (E) HMGB1 and (F) ATP levels. (G) Quantitative analysis of CRT expression from panel H. (H) CRT exposure in Hepa 1–6 cells quantified by flow cytometry at 24h.

The intracellular uptake efficiency of Dil-labeled NBs and Lipo3000 complexes by HepG2 cells was evaluated. As shown in Fig. 4A, Lipo3000 demonstrated more rapid cellular internalization than NBs at 4 h post-incubation, intense intracellular red fluorescence was observed, and flow cytometry showed an uptake rate of about 69 % for Lipo3000 versus 24 % for NBs (Fig. 4B–D). This accelerated uptake is likely attributable to the smaller nanoparticle size of the commercial Lipo3000 formulation, most likely *via* clathrin-mediated endocytosis or macropinocytosis. However, after 12 h of incubation, NB uptake increased to 87.8 %, closely approaching the 90.7 % uptake achieved with Lipo3000, implying that both carriers are eventually internalized at comparable levels given adequate incubation. NBs may enter cells through slower, possibly alternative endocytic pathways or even through passive accumulation and prolonged cell-surface association.

### 3.4 ICD induction *in vitro*

To investigate the ICD-inducing potential of miR-195-5p/SK-NBs, we quantified three classical ICD-associated DAMPs: cell-surface CRT exposure, extracellular ATP release, and HMGB1 secretion. HMGB1 secretion, a nuclear DAMP that activates TLR4 signaling and facilitates dendritic cell maturation, was significantly increased following miR-195-5p/SK-NB + US treatment (0.90 ± 0.01 ng/mL), compared to SK-NBs + US (0.83 ± 0.02 ng/mL), free SK (0.79 ± 0.01 ng/mL), and controls (0.68 ± 0.01 ng/mL) (Fig. 4E). Consistent with HMGB1 secretion, extracellular ATP quantification was greatest in the miR-195-5p/SK-NBs + US group (15.70 ± 0.25 μmol/mL), exceeding levels in SK-NBs + US (11.24 ± 0.22 μmol/mL), free SK (13.66 ± 0.48 μmol/mL), and controls (4.92 ± 0.23 μmol/mL). ATP serves as a potent chemoattractant for antigen-presenting cells, and its elevated release further supports the immunostimulatory potential of the NBs platform [29] (Fig. 4F). In addition, cell surface exposure of CRT represents a key DAMP that plays a critical role in dendritic cell maturation and antigen cross-presentation [30]. Flow cytometric analysis of Hepa 1–6 cells treated with various formulations revealed differential CRT expression profiles across groups (Fig. 4G and H). Compared to controls (9.40 ± 2.79 %), N-NBs + US (20.78 ± 1.21 %), and miR-195-5p-NBs + US (22.27 ± 0.96 %), SK-containing groups exhibited significantly elevated CRT exposure: SK (58.29 ± 1.58 %, *P* < 0.001), SK-NBs + US (75.29 ± 4.75 %, *P* < 0.0001), and miR-195-5p/SK-NBs + US (81.53 ± 4.34 %, *P* < 0.0001)**.** Collectively, these data demonstrate that SK-loaded NBs—especially those co-loaded with miR-195-5p—robustly trigger multiple ICD markers, likely due to synergistic pro-apoptotic and immunomodulatory actions. Moreover, the partial ICD-inducing effect observed in the N-NBs + US group highlights the potential of NBs-mediated cavitation alone to modulate ICD stress, further supporting the value of this platform in combination immunotherapy strategies.

### 3.5 Suppression of PD-L1 expression *in vitro*

**Fig. 5:**
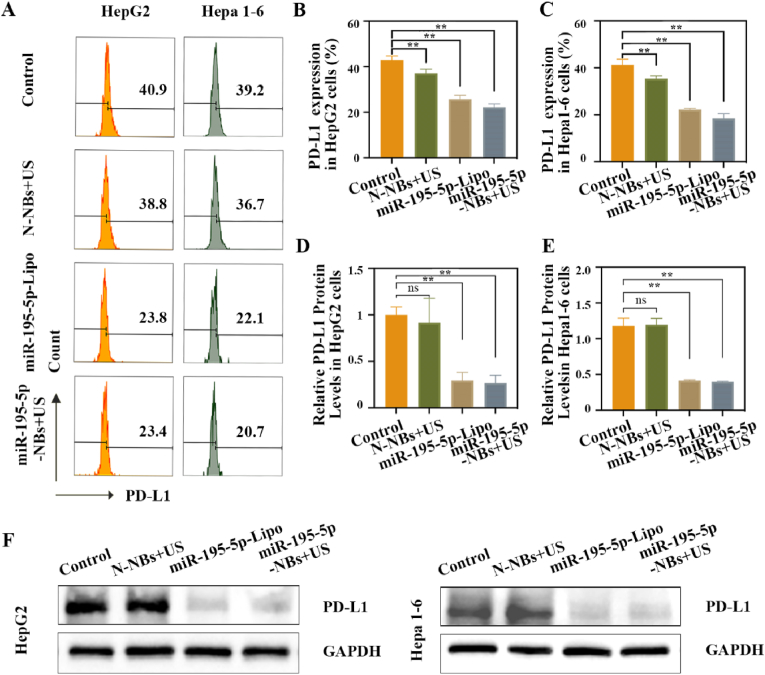
**miR-195-5p/SK-NBs-mediated suppression of PD-L1 expression *in vitro*.** Hepa 1–6 and HepG2 cells were treated with N-NBs + US, miR-195-5p-Lipo3000, and miR-195-5p-NBs + US for 48 h. (A) Flow cytometry histograms of surface PD-L1 expression. (B–C) Quantitative analysis of PD-L1 expression levels from panel C in (B) HepG2 cells and (C) Hepa 1–6 cells. (D–E) Quantification of PD-L1 protein levels from panel F in (D) HepG2 cells and (E) Hepa 1–6 cells. (F)Western blot of total PD-L1 protein expression.

To evaluate the effect of miR-195-5p-NBs and miR-195-5p-Lipo3000 on PD-L1 expression, both murine hepatoma Hepa 1–6 and human HCC HepG2 cells were treated with various formulations. Western blotting and flow cytometry were employed to assess PD-L1 expression, respectively. As shown in Fig. 5A–C, miR-195-5p-NBs + US and miR-195-5p-Lipo3000 reduced PD-L1 expression in both cell lines (*P* < 0.01 vs. control). Western blot analysis revealed a significant decrease in total PD-L1 protein expression in both cell lines following treatment with either miR-195-5p-Lipo3000 or miR-195-5p-NBs + US (Fig. 5D–F). Densitometric quantification showed that miR-195-5p-NBs + US reduced PD-L1 protein levels by ∼73 % in HepG2 and∼78 % in Hepa 1–6 cells, closely matching the suppression observed with miR-195-5p-Lipo3000 (∼70 % and ∼76 %, respectively). The cavitation effects generated during UTMD disrupt endosomal membranes, facilitating direct cytosolic delivery of miR-195-5p and resulting in transfection efficiency comparable to that of commercial reagents. In addition, NBs offer distinct advantages over conventional lipid-based transfection reagents. The NBs system enables spatiotemporally US-controlled miRNA release, and bypasses chemical transfection reagents, which are often associated with cytotoxicity, poor *in vivo* stability, and limited tumor penetration.

### 3.6 Distribution of miR-195-5p/SK-NBs *in vivo*

**Fig. 6:**
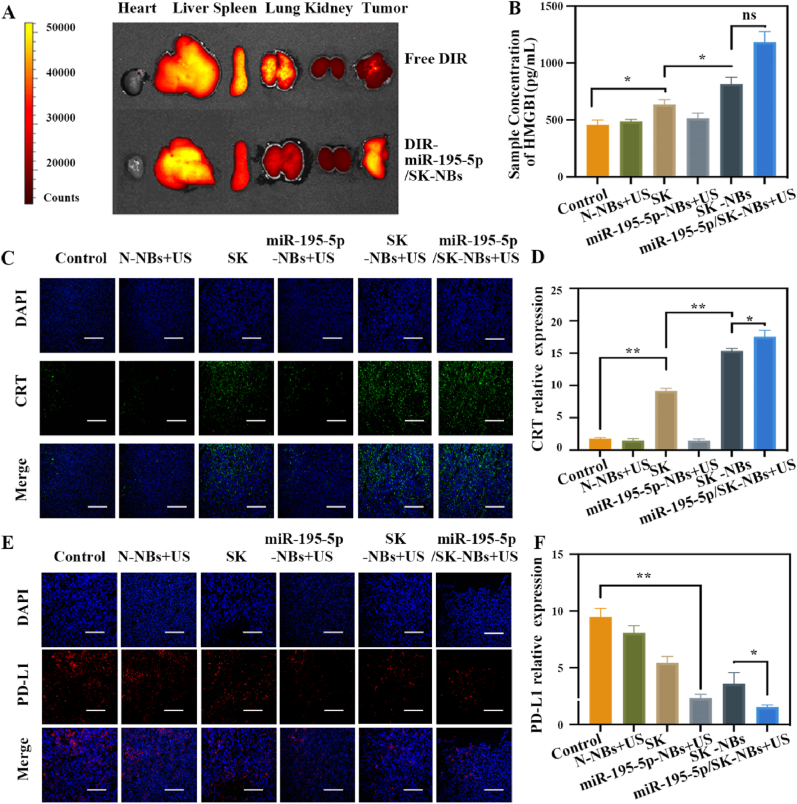
***In vivo* biodistribution and tumor analysis of ICD and PD-L1 protein** (A) *Ex vivo* fluorescence imaging of Dil and Dil-miR-195-5p/SK-NBs at 24 h post-injection. H22 xenograft–bearing mice were randomized into Control, N-NBs + US, free SK, miR-195-5p-NBs + US, SK-NBs + US, and miR-195-5p/SK-NBs + US. Mice were treated on days 1, 4, 7, 10, and 13. Then, tumors were harvested on 14 d for the following assays: (B) HMGB1 in tumor tissues. (C) Immunofluorescence staining of CRT in tumor tissues (scale bar = 100 μm). (D) Quantification of CRT fluorescence intensity from panel C. (E) Immunofluorescence staining of PD-L1 in tumor tissues (scale bar = 100 μm). (F) Quantitative analysis of PD-L1 fluorescence intensity from panel E.

The therapeutic feasibility was further validated through *in vivo* experiments. We first determined the optimal timing for US irradiation by assessing the tumor accumulation kinetics of miR-195-5p/SK-NBs. Mice bearing tumor xenografts were intravenously administered with either free Dil fluorescent dye or Dil-labeled miR-195-5p/SK-NBs. After 24 h, major organs (heart, liver, spleen, lungs, and kidneys) and tumor tissues were harvested for *ex vivo* fluorescence imaging analysis. It was observed a significant enhancement in tumor-specific fluorescence intensity in the miR-195-5p/SK-NBs group compared to free Dil controls, demonstrating superior tumor accumulation efficiency of the nanoliposomal formulation (Fig. 6A). This enhanced tumor accumulation can be attributed to the US-mediated drug delivery system, which capitalizes on both the EPR effect and US-induced cavitation dynamics, thereby spatiotemporally controlled drug release [31]. The nanoliposomal encapsulation confers dual protective advantages—shielding miR-195-5p and SK from circulatory nucleases/proteases and minimizing toxicity-associated adverse effects [32].

### 3.7 ICD induction *in vivo*

Building upon the promising *in vitro* ICD induction results, we further investigated ICD activation *in vivo*. Tumor tissues were harvested from each treatment group for quantitative analysis of ICD biomarkers: HMGB1 secretion levels were measured using an ELISA kit [33], while CRT expression was assessed through immunofluorescence staining. HMGB1 acts as a key DAMP molecule ICD, where its release from stressed or dying tumor cells facilitates dendritic cell maturation and antigen presentation *via* binding to Toll-like receptor 4 or receptor for advanced glycation end-products. This interaction triggers pro-inflammatory cytokine secretion (e.g., IL-1β, TNF-α) and enhances cross-priming of tumor-specific CD8^+^ T cells, thereby amplifying anti-tumor immunity [34,35]. It is revealed a 3.2-fold elevation in extracellular HMGB1 levels in the miR-195-5p/SK-NBs + US group (1184.17 ± 91.62 pg/mL) compared to controls (456.55 ± 43.36 pg/mL), with intermediate increases in SK (636.44 ± 43.07 pg/mL) and SK-NBs + US (816.99 ± 58.23 pg/mL) groups (Fig. 6B). Consistent with these findings, immunofluorescence analysis of CRT expression showed a similar pattern (Fig. 6C and D), where miR-195-5p/SK-NBs + US treatment induced a 5.1-fold increase in CRT + tumor cells (17.53 ± 1.00 % vs. 1.76 ± 0.15 % in controls), surpassing SK-NBs + US (15.34 ± 0.41 %) and SK (9.15 ± 0.40 %) monotherapies. These coordinated increases in HMGB1 secretion and CRT ectodomain exposure confirm robust ICD activation *in vivo*, positioning miR-195-5p/SK-NBs as a potent inducer of ICD.

### 3.8 In vivo modulation of PD-L1 expression by miR-195-5p

To validate the regulatory effect of miR-195-5p on PD-L1 expression *in vivo*, we performed immunofluorescence analysis of tumor tissues across treatment cohorts. Quantitative evaluation revealed a marked reduction in PD-L1 fluorescence intensity in the miR-195-5p-NBs + US (2.32 ± 0.33 AU, *P* < 0.01) and miR-195-5p/SK-NBs + US groups (1.54 ± 0.20 AU, *P* < 0.01) compared to control tumors (9.47 ± 0.75AU) (Fig. 6E and F). Importantly, the combinatorial miR-195-5p/SK-NBs treatment achieved synergistic suppression, surpassing the effects of individual components by 1.51-fold (vs. miR-195-5p-NBs alone). Spatial analysis further demonstrated PD-L1 reduction predominantly localized to tumor margins infiltrated by CD8^+^ T cells, suggesting microenvironmental crosstalk between PD-L1 modulation and immune cell recruitment [36]. These concordant *in vivo* and *in vitro* findings establish miR-195-5p as a robust PD-L1 regulator across biological scales, with therapeutic synergy emerging from its integration with SK. Upregulation of PD-L1 expression in tumor cells may enhance immune evasion by binding to PD-1 receptors on cytotoxic T lymphocytes, thereby suppressing anti-tumor immune responses [37].

### 3.9 In vivo immunoactivation

**Fig. 7:**
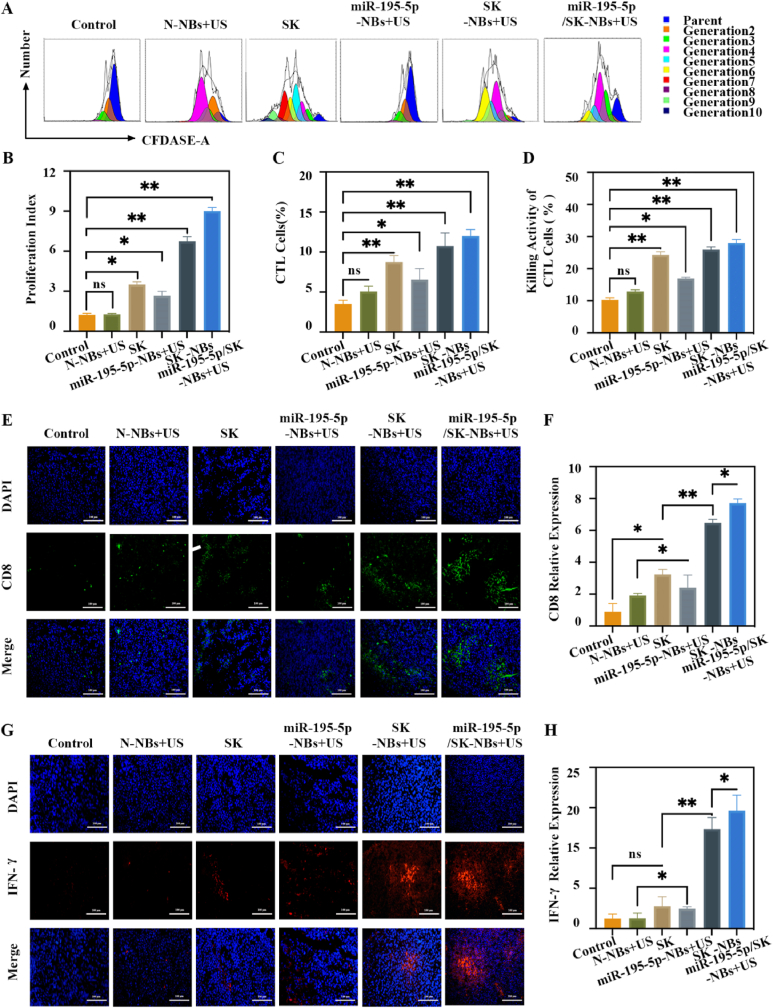
**Immune-promoting effects.** H22 xenograft–bearing mice were randomized into Control, N-NBs + US, free SK, miR-195-5p-NBs + US, SK-NBs + US, and miR-195-5p/SK-NBs + US. Mice were treated on days 1, 4, 7, 10, and 13 d. Then tumors and spleens were collected for immune profiling. (A) Flow cytometry analysis of splenic lymphocyte proliferation following different treatments. (B) Quantitative comparison of lymphocyte proliferation from panel A. (C) Flow cytometric quantification of cytotoxic T lymphocyte proportions. (D) LDH assay to assess CTL-mediated cytotoxicity. (E) Immunofluorescence staining of CD8^+^ T cells in tumor tissues (scale bar = 100 μm). (F) Quantification of CD8^+^ T cell infiltration from panel E. (G) Immunofluorescence staining of IFN-γ in tumor tissues (scale bar = 100 μm). (H) Quantitative analysis of IFN-γ expression from panel G.

It is demonstrated that miR-195-5p/SK-NBs not only promote ICD but also attenuate immune escape by downregulating PD-L1 expression *in vitro* and *in vivo*. This dual action highlights the therapeutic potential of this platform for combination immunotherapy. Thus, we systematically evaluated their capacity to activate antitumor immunity through multiple immunological assays. Splenic lymphocyte proliferation assays revealed that all treatment groups (SK, miR-195-5p-NBs + US, SK-NBs + US, and miR-195-5p/SK-NBs + US) exhibited enhanced proliferative activity compared to untreated controls (Fig. 7A and B). The miR-195-5p/SK-NBs + US group showed the most active *in vitro* proliferation, with a proliferation index approximately 7.30 times that of the control group (P < 0.01). Splenic lymphocyte proliferation serves as a critical indicator of systemic immune activation in cancer immunotherapy, reflecting the expansion of tumor-specific T and B cell populations. Enhanced proliferation correlates with improved antigen-specific effector functions, including cytotoxic CTL-mediated tumor cell killing and memory cell formation [38]. CTL profiling and functional analysis are pivotal in evaluating anti-tumor immune responses, as CTLs directly mediate tumor cell lysis through perforin/granzyme release and Fas/FasL interactions [39,40]. Subsequent CTL profiling *via* flow cytometry (Fig. 7C) showed significant elevations in CD8^+^ CTL frequencies within splenocytes from treated mice. The miR-195-5p/SK-NBs + US group exhibited the highest CTL expansion (12.00 ± 0.81 % vs. 3.50 ± 0.47 % in controls, *P* < 0.01), correlating with enhanced tumoricidal activity. Parallel LDH release assays (Fig. 7D) confirmed this functional enhancement: when co-cultured with H22 HCC cells, antigen-primed splenocytes from miR-195-5p/SK-NBs-treated mice demonstrated superior cytotoxicity (28.02 ± 1.09 % specific lysis at 50:1 effector-to-target ratio vs. 10.27 ± 0.68 % in controls, *P* < 0.01), outperforming single-modality treatments. To further dissect intratumoral immune modulation, immunofluorescence analysis of tumor sections (Fig. 7E–H) revealed coordinated upregulation of CD8^+^ T cell infiltration (8.72-fold increase in density vs. controls, *P* < 0.001) and IFN-γ production (11.66-fold elevation in fluorescence intensity, *P* < 0.01) in the miR-195-5p/SK-NBs cohort. These synergistic effects—systemic lymphocyte activation, CTL-mediated cytotoxicity, and tumor microenvironment reprogramming—collectively underscore the capacity of miR-195-5p/SK-NBs to orchestrate a multipronged antitumor immune response. The convergence of these immunological axes positions miR-195-5p/SK-NBs as a promising combinatorial immunotherapeutic platform for HCC, bridging innate ICD induction with adaptive immune potentiation. Building upon our prior success in UTMD-enabled combinatorial therapy—achieving tumor-specific soluble PD-1 (sPD-1) gene transfection coupled with chlorin e6 (Ce6)-mediated sonodynamic therapy to potentiate PD-1/PD-L1 blockade [41]—this study introduces a transformative advancement by integrating SK, a naphthoquinone-based ICD inducer, into the therapeutic paradigm. The SK-incorporated platform elicited profound remodeling of the TIME, as evidenced by an increase in CD8^+^ cytotoxic T lymphocyte infiltration density and IFN-γ production (9–12 fold vs. 3–5 fold), significantly surpassing the immunogenic effects of the original sPD-1/Ce6 strategy.

### 3.10 Antitumor capacity *in vivo*

**Fig. 8:**
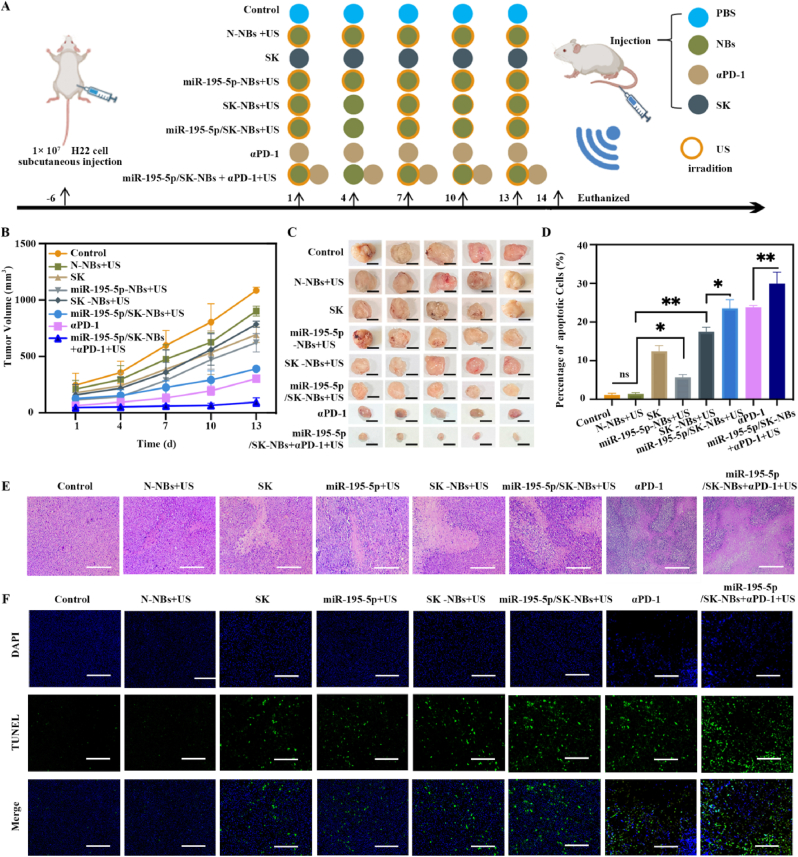
**Therapeutic efficacy *in vivo*.** (A) Schematic of the tumor treatment protocol. H22 tumor-bearing mice were randomly divided into eight groups and received tail vein injections on days 1, 4, 7, 10, and 13. Treatment groups included saline (Control), free SK, N-NBs, miR-195-5p-NBs, SK-NBs, miR-195-5p/SK-NBs, αPD-1, and αPD-1 + miR-195-5p/SK-NBs. For US-mediated groups (N-NBs + US, miR-195-5p-NBs + US, SK-NBs + US, miR-195-5p/SK-NBs + US, and miR-195-5p/SK-NBs + αPD-1 + US), tumors were locally irradiated with US (1 MHz, 1 W/cm^2^, 50 % duty cycle, 120 s) immediately after injection. (B) Tumor growth curves for each treatment group over the study period. (C) Representative images of excised tumors from each group at the study endpoint (scale bar = 0.5 cm). (D) Quantitative analysis of apoptotic cells from panel F. (E) H&E staining of tumor sections showing necrotic regions following different treatments (scale bar = 100 μm). (F) TUNEL assay to assess tumor cell apoptosis (scale bar = 100 μm).

As described above, miR-195-5p/SK-NBs effectively alleviated the TIME. To further explore their therapeutic potential, we investigated whether this formulation could enhance the antitumor efficacy of αPD-1 treatment. Fig. 8A depicts the *in vivo* treatment regimen. The detailed protocol is provided in Section 2.11. The tumor volumes of mice were measured during the treatment intervention period. As shown in Fig. 8B and C, it was observed that the control group exhibited the most aggressive tumor progression, with a mean tumor volume reaching 1085.95 ± 29.32 mm^3^ by day 13. Although miR-195-5p/SK-NBs exhibited strong immune activation by enhancing IFN-γ production and CD8^+^ T cell recruitment, this did not fully translate into superior antitumor efficacy *in vivo* (the mean tumor volume of the miR-195-5p/SK-NBs group is 390.51 ± 16.88 mm^3^). This discrepancy could be attributed to several factors, including the immunosuppressive tumor microenvironment that may impair the function of infiltrating T cells, or a potential delay in translating immune activation into measurable tumor regression. We therefore combined miR-195-5p/SK-NBs with αPD-1. Notably, the combination therapy led to the most pronounced antitumor effect (mean tumor volume: 94.57 ± 38.21 mm^3^, p < 0.01 versus control). miR-195-5p/SK-NBs trigger a strong local immune response by inducing ICD and reducing PD-L1 expression, yet residual checkpoint interactions still limit CD8^+^ T cell function. The addition of αPD-1 restores the full cytotoxic activity of primed T cells. Together with reduced PD-L1 expression, it also enhances dendritic cell–mediated priming of naïve T cells, promotes their proliferation and cytokine secretion, and maintains cytotoxic effector function. These combined effects transform early immune activation into a more robust and durable systemic antitumor response [42]. Similarly, Zhang et al. engineered a cationic liposomal formulation (CG-J/ZL) co-encapsulating the DNMT inhibitor zebularine, the BRD4 inhibitor JQ1, and the TLR9 agonist CpG. In their system, Zeb upregulated the expression of tumor-associated antigens to enhance tumor immunogenicity, JQ1 suppressed PD-L1 expression to interrupt the PD-1/PD-L1 immune checkpoint, and CpG promoted dendritic cell maturation. When combined with an αPD-1, CG-J/ZL produced a markedly enhanced antitumor effect [43].

Tumor tissues from each group of treated mice were collected and made into paraffin sections. Histopathological evaluation through H&E staining was performed to observe the necrosis in the tumor tissues, evaluating their antitumor effects. Control tumors displayed characteristic malignant features, including high cellular density, disordered architecture, and marked nuclear atypia. In contrast, treatment groups exhibited dose-dependent tumor necrosis, with the miR-195-5p/SK-NBs + αPD-1 + US group showing the most extensive necrotic areas, characterized by nuclear fragmentation, cytoplasmic eosinophilia, and large homogeneous regions of coagulative necrosis. These quantitative histopathological findings corroborate the therapeutic superiority of the combined treatment approach (Fig. 8D). To quantify treatment-induced apoptosis, we performed TUNEL assays on paraffin-embedded tumor sections. Apoptotic bodies were labeled with green fluorescence, and the number of apoptotic cells in each group was quantitatively analyzed using ImageJ software. Fig. 8E, F revealed that the control group exhibited minimal apoptosis (1.14 ± 0.42 % TUNEL-positive cells). In contrast, treatment groups showed significantly increased apoptotic indices: SK (12.45 ± 1.45 %), miR-195-5p-NBs + US (5.71 ± 0.72 %), SK-NBs + US (17.48 ± 1.15 %), miR-195-5p/SK-NBs + US (23.54 ± 2.24 %), αPD-1 (23.78 ± 0.51 %), and miR-195-5p/SK-NBs + αPD-1 + US (29.93 ± 2.92 %). The miR-195-5p/SK-NBs + αPD-1 + US group demonstrated a 26-fold increase in apoptotic cells compared to controls, indicating superior pro-apoptotic activity. Collectively, these results validate miR-195-5p/SK-NBs reverses immunosuppressive factors, priming the tumor microenvironment for enhanced αPD-1 response. It provides new insights into improving the clinical performance of αPD-1 therapy, particularly given its variable efficacy in patients. Furthermore, future studies will evaluate long‐term survival benefits in orthotopic and metastatic models.

### 3.11 Biosafety assay

**Fig. 9:**
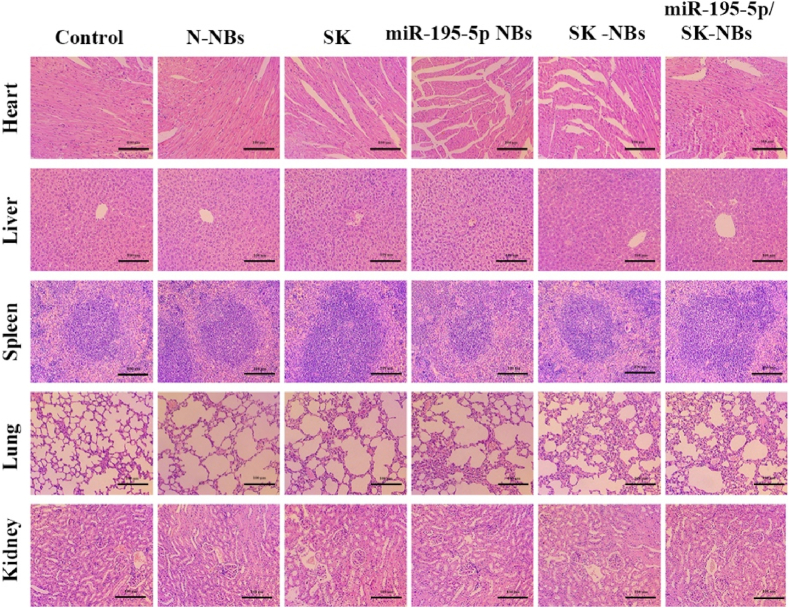
**Histopathological analysis of major organs to assess systemic toxicity.** At the end of the treatment cycle, major organs—including the heart, liver, spleen, lungs, and kidneys—were harvested from H22 tumor-bearing mice in all experimental groups. Tissues were stained with H&E to assess potential histopathological alterations indicative of systemic toxicity (scale bar = 100 μm).

The biosafety of NBs is a critical aspect of facilitating their clinical translation [44]. Organs from each group of mice were harvested and subjected to H&E staining to observe any histopathological changes and assess the safety of the drugs. As shown in Fig. 9, histopathological evaluation revealed preserved tissue architecture and normal cellular morphology across all treatment groups. Specifically, cardiac sections displayed intact myocardial fibers with no evidence of inflammatory infiltration or necrosis. Hepatic tissues maintained normal lobular architecture with no signs of steatosis or hepatocyte degeneration. Splenic sections showed preserved red and white pulp organization without evidence of lymphoid depletion. Pulmonary tissues exhibited normal alveolar structure and bronchiolar epithelium, while renal sections demonstrated intact glomerular and tubular architecture. These findings demonstrate that neither the individual components nor the combined miR-195-5p/SK-NBs formulation induced detectable organ toxicity at the administered doses, supporting their favorable safety profile for potential clinical translation.

## 4 Conclusion

This study establishes a novel US-activated NBs platform for the co-delivery of miR-195-5p and SK, designed to synergistically improve the therapeutic efficacy of αPD-1 immunotherapy in HCC. By leveraging UTMD, the platform achieves spatiotemporally controlled miR-195-5p release and transfected, downregulating PD-L1 expression to achieve immune checkpoint blockade, while concurrently enabling SK-mediated ICD *via* coordinated release of DAMPs, including HMGB1, CRT, and ATP. *In vivo*, miR-195-5p/SK-NBs reprogrammed the TIME *via* multifaceted immunomodulation: enhancing splenic lymphocyte proliferation, amplifying cytotoxic CD8^+^ T cell infiltration and IFN-γ secretion, and elevating CTL activity and proportion, collectively improving the treatment efficiency of αPD-1. The therapeutic superiority stems from the coordinated interplay between PD-L1 blockade and ICD-driven immune activation, which offers a promising avenue for HCC treatment.

## CRediT authorship contribution statement

**Yandi Tan:** Writing – review & editing, Investigation, Data curation. **Qiao Xu:** Writing – original draft, Software. **Yezi Chen:** Resources, Conceptualization. **Yun Liu:** Investigation. **Chaoqi Liu:** Validation. **Xinwu Cui:** Supervision, Methodology. **Yun Zhao:** Funding acquisition, Conceptualization.

## Funding

This work was supported by the 10.13039/501100001809National Natural Science Foundation of China (Grants:82371992) and the 10.13039/501100002858China Postdoctoral Science Foundation (Certificate Number:2024M751018).

## Declaration of competing interest

The authors declare that they have no known competing financial interests or personal relationships that could have appeared to influence the work reported in this paper.

## Data Availability

Data will be made available on request.

## References

[1] Siegel R.L., Giaquinto A.N., Jemal A. (2024). Cancer statistics. CA Cancer J. Clin..

[2] Llovet J.M., Kelley R.K., Villanueva A., Singal A.G., Pikarsky E., Roayaie S., Lencioni R., Koike K., Zucman-Rossi J., Finn R.S. (2021). Hepatocellular carcinoma. Nat. Rev. Dis. Primers.

[3] Minton K. (2023). Immune checkpoint blockade breaches the mucosal firewall to induce gut microbiota translocation. Nat. Rev. Immunol..

[4] Song F., Hu B., Liang X., Cheng J., Wang C., Wang P., Wang T., Tang P., Sun H., Guo W., Zhou J., Fan J., Chen Z., Yang X. (2024). Anlotinib potentiates anti‐PD1 immunotherapy via transferrin receptor‐dependent CD8^+^ t‐cell infiltration in hepatocellular carcinoma. Clin. Transl. Med..

[5] Ma M., Zhang Y., Pu K., Tang W. (2025). Nanomaterial-enabled metabolic reprogramming strategies for boosting antitumor immunity. Chem. Soc. Rev..

[6] Wu X., Li Y., Wen M., Xie Y., Zeng K., Liu Y.-N., Chen W., Zhao Y. (2024). Antitumor immunity: fabrication, mechanisms and applications. Chem. Soc. Rev..

[7] Yu J., Zhou B., Zhang S., Yin H., Sun L., Pu Y., Zhou B., Sun Y., Li X., Fang Y., Wang L., Zhao C., Du D., Zhang Y., Xu H. (2022). Design of a self-driven probiotic-CRISPR/Cas9 nanosystem for sono-immunometabolic cancer therapy. Nat. Commun..

[8] Galluzzi L., Guilbaud E., Schmidt D., Kroemer G., Marincola F.M. (2024). Targeting immunogenic cell stress and death for cancer therapy. Nat. Rev. Drug Discov..

[9] Luo M., Wang Y., Zhao F., Luo Y. (2025). Recent advances in nanomaterial‐mediated cell death for cancer therapy. Adv. Healthcare Mater..

[10] Linderman S.W., DeRidder L., Sanjurjo L., Foote M.B., Alonso M.J., Kirtane A.R., Langer R., Traverso G. (2025). Enhancing immunotherapy with tumour-responsive nanomaterials. Nat. Rev. Clin. Oncol..

[11] Yu Z., Gao J., Zhang X., Peng Y., Wei W., Xu J., Li Z., Wang C., Zhou M., Tian X., Feng L., Huo X., Liu M., Ye M., Guo D., Ma X. (2023). Correction: characterization of a small-molecule inhibitor targeting NEMO/IKKβ to suppress colorectal cancer growth. Signal Transduct. Targeted Ther..

[12] Krysko D.V., Garg A.D., Kaczmarek A., Krysko O., Agostinis P., Vandenabeele P. (2012). Immunogenic cell death and DAMPs in cancer therapy. Nat. Rev. Cancer.

[13] Li J., Zhou S., Yu J., Cai W., Yang Y., Kuang X., Liu H., He Z., Wang Y. (2021). Low dose shikonin and anthracyclines coloaded liposomes induce robust immunogenetic cell death for synergistic chemo-immunotherapy. J. Contr. Release.

[14] Li S., Zhang T., Xu W., Ding J., Yin F., Xu J., Sun W., Wang H., Sun M., Cai Z., Hua Y. (2020). Sarcoma-targeting peptide-decorated polypeptide nanogel intracellularly delivers shikonin for upregulated osteosarcoma necroptosis and diminished pulmonary metastasis: erratum. Theranostics.

[15] Chen J., Qiu S., Liu Y., Sun W., Zhou T., Zhao L., Li Z., Duan Y. (2024). Ultrasound targeted microbubble destruction assisted exosomal delivery of siHmox1 effectively inhibits doxorubicin-induced cardiomyocyte ferroptosis. J. Nanobiotechnol..

[16] Li X., Duan Z., Chen X., Pan D., Luo Q., Gu L., Xu G., Li Y., Zhang H., Gong Q., Chen R., Gu Z., Luo K. (2023). Impairing tumor metabolic plasticity via a stable metal‐phenolic‐based polymeric nanomedicine to suppress colorectal cancer. Adv. Mater..

[17] Wang X., Li F., Zhang J., Guo L., Shang M., Sun X., Xiao S., Shi D., Meng D., Zhao Y., Jiang C., Li J. (2024). A combination of PD-L1-targeted IL-15 mRNA nanotherapy and ultrasound-targeted microbubble destruction for tumor immunotherapy. J. Contr. Release.

[18] Liang J., Qiao X., Qiu L., Xu H., Xiang H., Ding H., Chen Y. (2024). Engineering versatile nanomedicines for ultrasonic tumor immunotherapy. Adv. Sci..

[19] Nittayacharn P., Abenojar E., Cooley M.B., Berg F.M., Counil C., Sojahrood A.J., Khan M.S., Yang C., Berndl E., Golczak M., Kolios M.C., Exner A.A. (2024). Efficient ultrasound-mediated drug delivery to orthotopic liver tumors – direct comparison of doxorubicin-loaded nanobubbles and microbubbles. J. Contr. Release.

[20] Xu Q., Xu J.-L., Chen W.-Q., Xu W.-X., Song Y.-X., Tang W.-J., Xu D., Jiang M.-P., Tang J. (2022). Roles and mechanisms of miR-195–5p in human solid cancers. Biomed. Pharmacother..

[21] Shadbad M.A., Safaei S., Brunetti O., Derakhshani A., Lotfinejad P., Mokhtarzadeh A., Hemmat N., Racanelli V., Solimando A.G., Argentiero A., Silvestris N., Baradaran B. (2021). A systematic review on the therapeutic potentiality of PD-L1-Inhibiting MicroRNAs for triple-negative breast cancer: toward single-cell sequencing-guided biomimetic delivery. Genes.

[22] Zhou W., Zhang M., Liu C., Kang Y., Wang J., Yang X. (2019). Long noncoding RNA LINC00473 drives the progression of pancreatic cancer via upregulating programmed death‐ligand 1 by sponging microRNA‐195‐5p. J. Cell. Physiol..

[23] Liu W., Chen H., Wang D. (2021). Protective role of astragaloside IV in gastric cancer through regulation of microRNA-195-5p-mediated PD-L1, Immunopharmacol. Immunotoxicol..

[24] Wang Q.-M., Lian G.-Y., Song Y., Huang Y.-F., Gong Y. (2019). LncRNA MALAT1 promotes tumorigenesis and immune escape of diffuse large B cell lymphoma by sponging miR-195. Life Sci..

[25] Yuan Y., Sun W., Xie J., Zhang Z., Luo J., Han X., Xiong Y., Yang Y., Zhang Y. (2025). RNA nanotherapeutics for hepatocellular carcinoma treatment. Theranostics.

[26] Wang Z., Chen J., Wang J., Xu M., Yang H., Yang H., Zhao C., Sun P., Ji H., Liu J., Shan J., Tian J., Li S., Yu D., Wang C., Yu X., Ding S., Xu W., Zhang Y., Leng X., R-Porter T. (2025). MSCs biomimetic ultrasonic phase change nanoparticles promotes cardiac functional recovery after acute myocardial infarction. Biomaterials.

[27] Guo C., Lin L., Wang Y., Jing J., Gong Q., Luo K. (2025). Nano drug delivery systems for advanced immune checkpoint blockade therapy. Theranostics.

[28] Chen Y., Luo X., Liu Y., Zou Y., Yang S., Liu C., Zhao Y. (2022). Targeted nanobubbles of PD-L1 mAb combined with doxorubicin as a synergistic tumor repressor in hepatocarcinoma. Int. J. Nanomed..

[29] Zhang Y., Fang Z., Pan D., Li Y., Zhou J., Chen H., Li Z., Zhu M., Li C., Qin L., Ren X., Gong Q., Luo K. (2024). Dendritic polymer‐based nanomedicines remodel the tumor stroma: improve drug penetration and enhance antitumor immune eesponse10.1002/adma.202401304. Adv. Mater..

[30] Meier P., Legrand A.J., Adam D., Silke J. (2024). Immunogenic cell death in cancer: targeting necroptosis to induce antitumour immunity. Nat. Rev. Cancer.

[31] Zeng Q., Li G., Chen W. (2023). Ultrasound-activatable and skin-associated minimally invasive microdevices for smart drug delivery and diagnosis. Adv. Drug Delivery Rev..

[32] Fatima M., Almalki W.H., Khan T., Sahebkar A., Kesharwani P. (2024). Harnessing the power of stimuli‐responsive nanoparticles as an effective therapeutic drug delivery system. Adv. Mater..

[33] Kuang J., Rao Z.-Y., Zheng D.-W., Kuang D., Huang Q.-X., Pan T., Li H., Zeng X., Zhang X.-Z. (2023). Nanoparticles hitchhike on monocytes for glioblastoma treatment after low-dose radiotherapy. ACS Nano.

[34] Yang M., Zhang C., Wang R., Wu X., Li H., Yoon J. (2023). Cancer immunotherapy elicited by immunogenic cell death based on smart nanomaterials. Small Methods.

[35] Demuynck R., Engelen Y., Skirtach A.G., De Smedt S.C., Lentacker I., Krysko D.V. (2024). Nanomedicine to aid immunogenic cell death (ICD)-based anticancer therapy. Trends Cancer.

[36] Diskin B., Adam S., Cassini M.F., Sanchez G., Liria M., Aykut B., Buttar C., Li E., Sundberg B., Salas R.D., Chen R., Wang J., Kim M., Farooq M.S., Nguy S., Fedele C., Tang K.H., Chen T., Wang W., Hundeyin M., Rossi J.A.K., Kurz E., Haq M.I.U., Karlen J., Kruger E., Sekendiz Z., Wu D., Shadaloey S.A.A., Baptiste G., Werba G., Selvaraj S., Loomis C., Wong K.-K., Leinwand J., Miller G. (2020). PD-L1 engagement on T cells promotes self-tolerance and suppression of neighboring macrophages and effector T cells in cancer. Nat. Immunol..

[37] Lee D., Cho M., Kim E., Seo Y., Cha J.-H. (2024). PD-L1: from cancer immunotherapy to therapeutic implications in multiple disorders. Mol. Ther..

[38] He X., Wang J., Tang Y., Chiang S.T., Han T., Chen Q., Qian C., Shen X., Li R., Ai X. (2023). Recent advances of emerging spleen‐targeting nanovaccines for immunotherapy. Adv. Healthcare Mater..

[39] Jiang M., Fang H., Tian H. (2025). Metabolism of cancer cells and immune cells in the initiation, progression, and metastasis of cancer. Theranostics.

[40] Viel S., Vivier E., Walzer T., Marçais A. (2025). Targeting metabolic dysfunction of CD8 T cells and natural killer cells in cancer. Nat. Rev. Drug Discov..

[41] Tan Y., Yang S., Ma Y., Li J., Xie Q., Liu C., Zhao Y. (2021). Nanobubbles containing sPD-1 and Ce6 mediate combination immunotherapy and suppress hepatocellular carcinoma in mice. Int. J. Nanomed..

[42] Kim T.K., Vandsemb E.N., Herbst R.S., Chen L. (2022). Adaptive immune resistance at the tumour site: mechanisms and therapeutic opportunities. Nat. Rev. Drug Discov..

[43] Liang S., Liu M., Mu W., Gao T., Gao S., Fu S., Yuan S., Liu J., Liu Y., Jiang D., Zhang N. (2024). Nano‐regulator inhibits tumor immune escape via the “two‐way regulation” epigenetic therapy strategy. Adv. Sci..

[44] Wang M., Yu F., Zhang Y. (2025). Present and future of cancer nano-immunotherapy: opportunities, obstacles and challenges. Mol. Cancer.

